# Wnt5a Signaling in Gastric Cancer

**DOI:** 10.3389/fcell.2020.00110

**Published:** 2020-03-04

**Authors:** Pablo Astudillo

**Affiliations:** Instituto de Ciencias Biomédicas, Facultad de Ciencias de la Salud, Universidad Autónoma de Chile, Santiago, Chile

**Keywords:** Wnt5a, gastric cancer, Disheveled, adhesion, invasion, metastasis, mechanosensing

## Abstract

Gastric cancer remains an important health challenge, accounting for a significant number of cancer-related deaths worldwide. Therefore, a deeper understanding of the molecular mechanisms involved in gastric cancer establishment and progression is highly desirable. The Wnt pathway plays a fundamental role in development, homeostasis, and disease, and abnormal Wnt signaling is commonly observed in several cancer types. Wnt5a, a ligand that activates the non-canonical branch of the Wnt pathway, can play a role as a tumor suppressor or by promoting cancer cell invasion and migration, although the molecular mechanisms explaining these roles have not been fully elucidated. Wnt5a is increased in gastric cancer samples; however, most gastric cancer cell lines seem to exhibit little expression of this ligand, thus raising the question about the source of this ligand *in vivo*. This review summarizes available research about Wnt5a expression and signaling in gastric cancer. In gastric cancer, Wnt5a promotes invasion and migration by modulating integrin adhesion turnover. Disheveled, a scaffolding protein with crucial roles in Wnt signaling, mediates the adhesion-related effects of Wnt5a in gastric cancer cells, and several studies provide growing support for a model whereby Disheveled-interacting proteins mediates Wnt5a signaling to modulate cytoskeleton dynamics. However, Wnt5a might induce other effects in gastric cancer cells, such as cell survival and induction of gene expression. On the other hand, the available evidence suggests that Wnt5a might be expressed by cells residing in the tumor microenvironment, where feedback mechanisms sustaining Wnt5a secretion and signaling might be established. This review analyzes the possible functions of Wnt5a in this pathological context and discusses potential links to mechanosensing and YAP/TAZ signaling.

## The Wnt Signaling Pathway: an Overview

The Wnt signaling pathway plays fundamental roles in the context of embryonic development and adult homeostasis (Logan and Nusse, 2004). This pathway is composed of a family of secreted Wnt ligands, Frizzled receptors, and several co-receptors, intracellular adaptors, and scaffolding proteins, and is commonly divided into two main branches.

The “canonical” (or “Wnt/β-catenin”) pathway depends on the stabilization of β-catenin, a protein that acts both at cell-cell interactions and as a transcription factor (Valenta et al., 2012). In the absence of a canonical Wnt signal, the pathway is inactivated through a negative feedback mechanism, which includes both a β-catenin destruction complex, which targets β-catenin for proteasomal degradation, and the plasma membrane ZNRF3/RNF43 ubiquitin ligases. Upon binding of a canonical Wnt ligand to a Frizzled receptor, several processes are triggered at the plasma membrane. First, binding of canonical Wnt ligands to Frizzled receptors leads to phosphorylation of the cytoplasmic domain of LRP5/6. Subsequently, intracellular components translocate to the cytosolic side of the membrane, followed by clustering and formation of a so-called “signalosome” (Davidson et al., 2005; Zeng et al., 2005; Gammons and Bienz, 2018). These events lead to direct inhibition of GSK-3β and internalization of the signalosome (Taelman et al., 2010). Consequently, the β-catenin degradation complex becomes inhibited, and newly synthesized β-catenin can translocate to the nucleus to exert its transcriptional role (Li et al., 2012). Other ligands and proteins also modulate this pathway. For instance, secreted R-spondin proteins have been shown to cooperate with Wnt ligands, potentiating the activation of the pathway (Binnerts et al., 2007; Wei et al., 2007; Kim et al., 2008). R-spondins bind to LGR4/5/6 receptors (de Lau et al., 2011; Glinka et al., 2011), leading to inactivation of the ZNRF3/RNF43 ubiquitin ligases and stabilization of Frizzled receptors (Hao et al., 2012; Koo et al., 2012).

On the other hand, there is a collection of “non-canonical” Wnt signaling pathways, which are independent of β-catenin stabilization. These non-canonical pathways also depend on the activation of intracellular proteins, including small GTPases RhoA, Rac1, and Cdc42, which activate downstream effectors such as DAAM1 and JNK (Schlessinger et al., 2009). Non-canonical Wnt signaling also depends on Wnt ligands, Frizzled receptors, and several co-receptors. These co-receptors include Ror1/2, Strabismus, Ryk, Vangl2, and heparan sulfate proteoglycans, among others (Niehrs, 2012). Depending on the available receptors, co-receptors, and intracellular effectors, several non-canonical pathways are defined (Semenov et al., 2007). Among these, two well-studied pathways are the planar cell polarity (Wnt/PCP) pathway and the Wnt/Ca^2+^ pathway (Veeman et al., 2003). Collectively, non-canonical Wnt pathways modulate different aspects of cell behavior, such as cell shape and migration. Of note, Disheveled acts as an intracellular scaffolding protein with roles in both canonical and non-canonical pathways (reviewed by Sharma et al., 2018).

Wnt ligands are typically classified as either canonical or non-canonical. This classification initially stemmed from the ability of ligands to induce the transformation of the C57MG cell line (Shimizu et al., 1997; Chien et al., 2009). For instance, Wnt3a and Wnt1 are classified as canonical ligands, whereas and Wnt5a and Wnt11 are considered non-canonical ligands. However, the specific pathway activated by a given Wnt ligand depends on the cellular context, such as the availability of co-receptors. Wnt5a provides an outstanding example of this complexity: besides activating non-canonical Wnt signaling, Wnt5a can both activate and repress Wnt/β-catenin signaling (Torres et al., 1996; Mikels and Nusse, 2006).

Given the multiple processes that follow Wnt stimulation, signaling levels must be tightly regulated, and proper termination mechanisms must be employed to dampen the signal when needed. In the case of the Wnt/β-catenin pathway, it is well known that several Wnt target genes (such as *AXIN2*, *NAKED1*, and *DKK1*) encode for proteins that inhibit the pathway, thus providing a negative feedback loop (Filipovich et al., 2011). Therefore, activating mutations in Wnt components, as well as persistently high levels of Wnt ligands, might lead to the establishment of diseases, including cancer (Logan and Nusse, 2004).

In this regard, the relationship of the Wnt/β-catenin pathway with cancer is well established, and several excellent reviews have been published recently (Anastas and Moon, 2013; Zhan et al., 2017). However, the role of the non-canonical Wnt pathway and its ligands in cancer is somewhat less understood. Once again, a remarkable example is Wnt5a. This ligand has been detected in several cancer types, and it is believed to play an essential role in both cancer cell migration and tumor suppression. However, the precise molecular role of Wnt5a in cancer has not been fully clarified. Therefore, a deeper understanding of the biological functions of Wnt5a in cancer is highly desirable. Comprehensive overviews of Wnt5a in cancer have been published elsewhere (Kikuchi et al., 2012; Endo et al., 2015; Asem et al., 2016). Instead, this review focuses on the specific role of Wnt5a in gastric cancer.

## Wnt5A Expression in Gastric Cancer

Gastric cancer (GC) is typically diagnosed at advanced stages, impairing adequate treatment, and leading to elevated mortality rates (Van Cutsem et al., 2016). An estimated more than 780,000 people died from stomach cancer in 2018, accounting for 8% of total cancer-related deaths, according to the Global Cancer Observatory (Bray et al., 2018). Therefore, GC remains a significant public health issue. Approximately 90% of all GC correspond to adenocarcinomas, which in turn can be classified as either diffuse- of intestinal-type, following the Lauren classification (Lauren, 1965; reviewed in Correa et al., 2009). Molecular criteria can be employed for identifying different subgroups, and some genetic abnormalities have been associated to GC (reviewed in detail by Van Cutsem et al., 2016; Ajani et al., 2017). Overall, gastric adenocarcinoma is conceived as a multistep process, sometimes referred to as the “Correa cascade” (Correa, 1988), where intestinal-type gastric cancers are preceded by a series of precancerous lesions, starting from gastritis, leading to metaplasia and invasive carcinoma (reviewed in Correa et al., 2009). In this regard, *Helicobacter pylori* infection constitutes a risk factor, due to induction of inflammation in the gastric mucosa. At later stages, epithelial gastric adenocarcinomas evolve to invasive lesions, where cancerous cells invade through the basement membrane and penetrate the mucosa and submucosa. Diffuse-type GC is also characterized by the stiffening of the gastric wall, or “linitis plastica” (Carneiro and Lauwers, 2012). Of note, other classifications for gastric adenocarcinomas have been proposed (reviewed in Carneiro and Lauwers, 2012), and other non-epithelial gastric tumors can also be identified (Wall and Nickl, 2019).

Given the role of Wnt signaling in cancer, several studies have analyzed the expression of Wnt ligands in GC. *WNT5A* mRNA was first shown to be up-regulated in samples of primary GC, compared to normal samples (Saitoh et al., 2002), and subsequent reports confirmed this observation. Kurayoshi and coworkers used GC tissue samples and matched non-neoplastic mucosa, reporting a 2.6-fold up-regulation of *WNT5A* expression by using semi-quantitative PCR (Kurayoshi et al., 2006). More recently, a bioinformatic meta-analysis of published transcriptomic data reported *WNT5A* expression in 617 out of 1,034 GC patients (Nam et al., 2014). At the protein level, Kurayoshi and coworkers reported Wnt5a expression in 30% of GC cases analyzed by immunohistochemical staining (Kurayoshi et al., 2006).

At the histological level, *WNT5A* is up-regulated in both intestinal-type (IGC) and diffuse-type (DGC) GC samples (Kurayoshi et al., 2006). Another study, performed with samples from Australian and Chinese patients, also showed significant up-regulation of *WNT5A* in both IGC and DGC (Boussioutas et al., 2003). More recently, Li and coworkers detected significant up-regulation of *WNT5A* in 21 out of 36 GC samples analyzed (Li et al., 2014). Of note, six out of the 36 samples showed down-regulation of *WNT5A* in this study. Therefore, it remains possible that GC might proceed following not only Wnt5a overexpression but also impaired or unbalanced Wnt ligand availability. Finally, high Wnt5a expression is correlated with poor prognosis (Kurayoshi et al., 2006), and there is a statistically significant correlation between Wnt5a expression and several clinical parameters, such as lymph node metastasis and tumor depth (Nam et al., 2017).

Collectively, these reports indicate that Wnt5a is highly abundant in GC, and likely plays a role in GC establishment and progression. However, the precise source of Wnt5a is unclear. While most reports cited previously employed GC samples for mRNA expression analysis or histological staining, the analysis of GC cell lines shows mixed results. Several GC cell lines have been used and characterized for *in vitro* studies. Although most of these cell lines share the same ethnicity, they differ in origin and histological type, in their expression profile for growth factors and cell cycle regulators, and their profile for common oncogenes (Yokozaki, 2000). Although genetic alterations for β-catenin and APC have not been reported in some of these cell lines, mutations in these genes have been described in AGS, MKN-28, and MKN-74 cells (Ikenoue et al., 2002; Ebert et al., 2003).

The expression of Wnt5a in GC cell lines is variable. For instance, Saitoh et al. (2002) reported undetectable levels of *WNT5A* in MNK-7 cells and low levels in MKN-45 cells; in contrast, Kurayoshi et al. (2006) reported an opposite expression profile. Both studies report undetectable levels of *WNT5A* in several GC cell lines, such as TMK-1, MKN-28, MKN-74, and KATO-III. On the other hand, Kanzawa and coworkers also observed Wnt5a protein expression in several GC cell lines, including MKN-7 cells (Kanzawa et al., 2013), while Zhao and coworkers analyzed Wnt5a expression in five GC cell lines, detecting it only in the MKN-45 cell line (Zhao et al., 2013). Miwa and coworkers also reported variable expression levels of *WNT5A* across a panel of GC cell lines (Miwa et al., 2017). Finally, Nam and coworkers observed variable Wnt5a protein expression in several GC cell lines, including the commonly used MKN-1, MKN-45, AGS, and NCI-N87 (Nam et al., 2014). Of note, the same report shows that Wnt3a, a prototypical canonical ligand, only has limited expression in a few GC cell lines.

Therefore, the evidence suggests that *WNT5A* might be poorly expressed in GC cell lines, thus raising the question about the source of this ligand in GC. One possibility is that Wnt5a might be expressed by cells residing in the tumor stroma. For instance, cancer-associated myofibroblasts (CAMs, also known as cancer-associated fibroblasts, or CAFs) from GC consistently displayed increased expression of Wnt5a, compared to adjacent tissue myofibroblasts (ATMs) (Wang et al., 2016). More recently, a study comparing the methylation and trimethylation (H3K23me3) patterns between CAFs and non-CAFs also identified *WNT5A* as a target for H3K23me3 predominantly in CAFs and corroborated that CAFs secreted more *WNT5A* than GC cell lines (Maeda et al., 2019). More importantly, this report showed that, in histological samples, *WNT5A* had higher expression in fibroblasts (which were identified by α-SMA staining), compared to cancer cells (Maeda et al., 2019).

Wnt5a might also be secreted by tumor-associated macrophages (TAMs), which are observed in GC and correlate with poor prognosis (Räihä and Puolakkainen, 2018). Macrophages secrete Wnt5a in response to LPS treatment and *H. pylori* infection (Zhao et al., 2013). In turn, Wnt5a might induce the recruitment of immune cells, sustaining an environment favorable for non-canonical Wnt signaling. One report showed that Wnt5a induces the expression of MCP-1 (monocyte chemotactic protein 1, also known as C-C Motif Chemokine Ligand 2, CCL2), a protein involved in macrophage recruitment (reviewed by Yoshimura, 2017), in two GC cell lines (BGC-803 and HGC-27) (Li et al., 2014). This report also showed that conditioned medium from Wnt5a-treated GC cells induced macrophage migration in Transwell assays, and this effect was lost after a neutralizing MCP-1 antibody was added to the medium, providing further support to a functional relationship between Wnt5a and MCP-1 in GC. In addition, *WNT5A* is correlated with *IL1B* and *CCL2* expression in GC tissues (Li et al., 2014). In this same line, it has been reported that Wnt5a is expressed by Cxcr4^+^ intraepithelial gastric innate lymphoid cells (ILCs) located in the isthmus, where the authors describe the existence of Mist1^+^ stem cells (Hayakawa et al., 2015). Wnt5a enhances the colony formation ability of these Mist1^+^ cells and, more importantly, cancer progression is impaired when Wnt5a expression is eliminated in the ILCs in a transgenic mouse model that develops diffuse-type GC following E-Cadherin depletion (Hayakawa et al., 2015).

Alternatively, Wnt5a might be specifically expressed in response to certain signals, such as environmental conditions or the interaction with stromal cells. In the first case, *H. pylori* infection has been linked to Wnt5a expression since *H. pylori* eradication leads to lower Wnt5a levels (Matsuo et al., 2012). Li et al. (2014) also reported significant upregulation of *WNT5A* in samples from *H. pylori*-positive patients compared to *H. pylori*-negative cases. On the other hand, MKN-7 cells (which, as mentioned above, exhibit low to moderate expression of Wnt5a) co-cultured with bone marrow mesenchymal stem cells show increased levels of *WNT5A* (Nishimura et al., 2012).

In conclusion, the discrepancy between high Wnt5a expression in GC biopsies or tissue arrays and modest or low expression observed in some GC cell lines can be explained by the presence of stromal cells or other inflammatory and environmental signals. Moreover, the evidence analyzed above suggests that GC cells have diverse sources of Wnt5a in the tumor microenvironment. In consequence, Wnt5a might be a suitable clinical target, at least in a subset of GC cases, fostering interest for studies addressing the molecular roles of Wnt5a in GC, which remain to be fully understood, as discussed below.

## Wnt5A in Gastric Cancer: Molecular Mechanisms

The role of Wnt5a has been studied in several cancer types, providing valuable insights into the possible mechanisms by which Wnt5a might influence cancer cell behavior. Wnt5a can function either as a regulator of cell migration and invasion or as a tumor suppressor (for two extensive reviews, see Kikuchi et al., 2012; Endo et al., 2015), leading to the notion of complex and opposing roles of this ligand in cancer (Pukrop and Binder, 2008; McDonald and Silver, 2009). Regarding the role of Wnt5a specifically in GC, it must be noted that overexpression of Wnt5a during adulthood failed to induce tumor initiation (Bakker et al., 2012), thus suggesting that Wnt5a alone is not sufficient for gastric cancer establishment. In addition, induction of Wnt5a overexpression from E13.5 during mouse development affected the intestinal tract; however, the stomach was not reported to be altered (van Amerongen et al., 2012). On the other hand, the tumor suppressor role of Wnt5a is commonly associated with low expression of this ligand (McDonald and Silver, 2009). The high expression of Wnt5a in GC, the lack of tumor initiation after overexpression in mice, and the evidence that will be reviewed in this section, collectively suggest that the predominant function of Wnt5a in GC is the regulation of cell migration and invasion.

Cell adhesion to the extracellular matrix (ECM) depends on integrin receptors and a complex set of intracellular proteins, which collectively form integrin-adhesion complexes (IACs) (Horton et al., 2016). In turn, cell migration and mechanotransduction depend on proper modulation of integrins and IAC proteins; therefore, signals able to modulate IAC dynamics are likely to be involved in cell migration and invasion, particularly in the context of cancer (Hamidi and Ivaska, 2018). Wnt5a stimulates cell migration in GC cell lines, whereas Wnt5a knockdown suppresses cell migration and invasion in Matrigel (Kurayoshi et al., 2006). Importantly, the relative migration of GC cell lines correlated with their relative levels of *WNT5A*, while the effect of Wnt5a was reduced by the negative Wnt regulator sFRP2 and by an anti-Wnt5a antibody, thus confirming the specificity of Wnt5a. This report also showed that Wnt5a increased MKN-45 cell adhesion, suggesting the involvement of IACs. In agreement with this observation, the abrogation of Wnt5a expression decreased the rate of assembly and disassembly of GFP-paxillin positive adhesions in MKN-1 cells. Of note, Wnt5a induced FAK Tyr397 phosphorylation and Rac activation in an Src- and PKC-dependent manner (Kurayoshi et al., 2006).

By promoting IAC dynamics, Wnt5a might lead to enhanced cell invasion. Supporting this idea, the injection of GC cell lines (KKLS and TMK-1) with reduced Wnt5A expression has less metastatic activity *in vivo* (Yamamoto et al., 2009). Furthermore, the treatment of GC cells with a polyclonal Wnt5a antibody (pAb5a-5) decreased cell adhesion, migration, and invasion of GC cell lines *in vitro*, and reduced the metastatic activity of the GC cell line KKLS *in vivo* (Hanaki et al., 2012).

Mechanistically, the Wnt5a antibody blocked Wnt5a- and clathrin-dependent internalization of Frizzled-2 and Ror2 receptors (Hanaki et al., 2012). A subsequent report from this group corroborated these observations using a second antibody targeting Wnt5a (mAb5A16), which reduced endocytosis of Ror-2 and Frizzled-2 and decreased the metastatic activity of the GC cell line KKLS *in vivo* (Shojima et al., 2015). On the other hand, a recent report unveiled the involvement of Ryk, another non-canonical Wnt receptor, in GC (Fu et al., 2019). The authors showed that Ryk is expressed in GC cell lines and samples from GC patients, correlating with clinical parameters such as wall invasion and liver metastasis. In addition, Ryk was shown to be required for migration and invasion in scratch and transwell assays (Fu et al., 2019). Of note, Ryk interacts with Vangl2, another non-canonical Wnt receptor, and Ryk knockdown impaired cell attachment. Collectively, these results suggest that non-canonical Wnt receptors are required for Wnt5a to modulate GC cell migration and invasion.

In order to modulate cell adhesion, Wnt5a must be able to functionally interact with intracellular effectors. The scaffolding protein Disheveled (Dvl) is a crucial effector in signaling through both canonical and non-canonical Wnt pathways. Dvl is required for HeLa S3 cells to remodel their focal adhesions, while Dvl knockdown impaired FAK Tyr397 phosphorylation and adhesion to fibronectin and collagen (Matsumoto et al., 2010). Mechanistically, Dvl localized to the cell periphery upon stimulation with Wnt5a, forming a complex with Frizzled-2 and APC, and interacting with FAK (Matsumoto et al., 2010). More importantly, Wnt5a required the Dvl-APC interaction to promote adhesion dynamics. The authors proposed a model whereby Wnt5a, Frizzled-2, and Disheveled form a complex proximal to integrins, FAK, and Paxillin, promoting adhesion dynamics through microtubule stabilization (Matsumoto et al., 2010).

A crucial aspect of Wnt signaling is the ability of Dvl to interact with other proteins (Sharma et al., 2018). A Dvl-interacting protein that is involved in Wnt5a-mediated adhesion dynamics is Daple (*Dvl-associating protein with a high frequency of leucine residues*). Daple promotes the interaction between Dvl and the PKCλ isoform of aPKC (Ishida-Takagishi et al., 2012), thus linking Dvl with the induction of FAK Tyr397 phosphorylation by Wnt5a (Kurayoshi et al., 2006). Daple was required for Dvl and PKCλ localization at the leading edge in migrating cells, whereas the Dvl-Daple interaction was also shown to be required for Rac1 activation and modulation of cell migration by Wnt5a. However, the precise molecular mechanism was not completely elucidated in this report (Ishida-Takagishi et al., 2012). The authors proposed a model whereby Daple allows the formation of a Dvl-PKCλ complex at the leading edge of Wnt5a-induced migrating cells, thus promoting Rac activation and actin reorganization.

Although Ishida-Takagishi et al. (2012) mainly used Vero and HEK293T cells in their study, evidence from a subsequent report provided further support for a role of Daple in GC. Daple was shown to be highly expressed in GC tumors, correlating with pathological characteristics (depth of gastric wall invasion, frequency of lymph node metastasis, and clinical stage), Wnt5a/b expression, and metastasis in xenograft tumor assays (Ara et al., 2016). More importantly, Daple knockdown impaired Wnt5a-induced Rac1 and JNK activation, decreased laminin γ2 expression in MKN-45 cells and attenuated MKN-45 and KKLS cell invasion in Matrigel assays, as well as the metastatic potential of KKLS cells *in vivo* (Ara et al., 2016). In consequence, Daple is likely to play a significant role in Wnt5a signaling in GC.

Another example of a Dvl-interacting protein is given by the microtubule (MT)-associated proteins Map7/7D1. Map7/7D1 binds to Dvl and modulates MT remodeling in HeLa cells, whereas Map7/7D1 knockdown impaired MT plus-end cortical targeting and focal adhesion turnover. Furthermore, Map7/7D1 promotes the cortical targeting of Dvl in response to Wnt5a, providing further support to the role of Dvl in cell migration dynamics during Wnt5a signaling (Kikuchi et al., 2018). However, it must be stressed that the specific relevance of these findings for gastric cancer remains to be fully explored.

Two additional Dvl-interacting proteins that have been associated with GC are Dapper homologs 1 (DACT1) and 2 (DACT2). DACT1 interacts with Dvl and induces its degradation and antagonizes canonical Wnt signaling (Zhang et al., 2006). DACT1 is also required for non-canonical Wnt/PCP signaling (Wen et al., 2010). Of note, *DACT1* levels are abrogated in several GC cell lines by hypermethylation, whereas DACT1 protein abundance is reduced in GC tissues compared to adjacent non-tumor tissue (Wang et al., 2012). Relevantly, *DACT1* promoter methylation was correlated with tumor size and metastasis (Wang et al., 2012). On the other hand, *DACT2* is also methylated in several GC cell lines, while protein levels are lower in GC tissue relative to normal mucosa (Yu et al., 2014). Of note, restoring DACT2 expression abrogated migration and invasion of the GC cell line SGC-7901 and tumor growth in a xenograft assay (Yu et al., 2014). Although the precise relationship of DACT1/2 with Wnt5a signaling was not addressed in these reports, Wnt5a might also influence GC by modulating these proteins. It remains possible that fine-tuned levels of these proteins might be required for proper Wnt5a signaling, and either gain or loss of expression might lead to imbalances and abnormal signaling.

The involvement of Dvl in Wnt5a-mediated adhesion dynamics is highly significant since this protein is at the crossroads of the canonical and non-canonical Wnt signaling pathways. The proper role of Dvl likely differs across tissues, depending on the balance between canonical and non-canonical Wnt signals, the availability of specific receptors and co-receptors, and the expression of Dvl-interacting proteins. Nevertheless, lessons from studies about Disheveled and Wnt5a in GC might shed light on the mechanisms of Wnt signaling in other cancer types. On the other hand, considering the high number of Dvl-interacting proteins (reviewed in Sharma et al., 2018), there is still much to be learned from Dvl in GC.

In addition to Dvl-mediated mechanisms, Wnt5a might also promote other intracellular responses, such as the expression of secreted proteins. For instance, Wnt5a was shown to promote the expression of laminin γ2, a component of the basement membrane protein laminin-5 (Yamamoto et al., 2009). This effect of Wnt5a is exerted through an AP-1 responsive element found in the *LAMC2* promoter and requires JNK and PKC activation, as well as the Frizzled-2 receptor. Knockdown of *LAMC2* decreased the invasive activity of TMK-1 and MKN-1 cells (the two cell lines in the study where *WNT5A* abundance best correlated with that of *LAMC2*), whereas Wnt5a showed a tendency to correlate with laminin γ2 expression in scirrhous GC biopsies (Yamamoto et al., 2009). Further research showed that the treatment of GC cells with the pAb5a-5 antibody impaired laminin γ2 expression and confirmed that TMK-1 cells with reduced expression of laminin γ2 exhibited decreased metastatic potential (Hanaki et al., 2012). Hence, Wnt5a might modulate the deposition of basement membrane proteins and other components of the extracellular matrix, including laminin γ2, thus promoting tumorigenesis by providing a favorable environment for GC progression and responsiveness to other secreted ligands. Finally, Wnt5a might also activate other signaling effectors. For instance, Liu and coworkers have reported that Wnt5a activates PI3K/Akt signaling in the GC cell line SGC-7901, resulting in GSK-3β phosphorylation and RhoA activation, promoting cell migration (Liu et al., 2013).

Other studies report additional effects that Wnt5a might play during GC establishment. For instance, Hayakawa et al. (2015) showed that Wnt5a activates RhoA in AGS and KATO-III cells, and increased the survival of E-Cadherin-deficient Mist1^+^ cells *in vitro*, in a mechanism involving RhoA. Wnt5a might also influence the interaction between GC cells and their tumor microenvironment (TME), contributing to a supportive environment for migration and invasion of transformed GC cells. In agreement with this idea, it has been reported that Wnt5a/Ror2 signaling in MSCs promotes the secretion of CXCL16, which requires the CXCR6 receptor on MKN-45 cells to induce proliferation (Takiguchi et al., 2016). Of note, MSCs express high levels of *ROR2* and *WNT5A* compared to MKN-45 cells, and abrogation of *ROR2* or *WNT5A* expression in MSCs impaired MSC-induced MKN-45 cell proliferation. Therefore, this report adds a new role for Wnt5a, namely the induction of cell proliferation *via* the CXCR6/CXCL16 axis. Considering the known role of CXCL16 in other cancers (Deng et al., 2010), the abovementioned data highlight the multiple functions that might be exerted by Wnt5a during GC.

Inflammation is another context where Wnt5a likely plays an important role. As mentioned above, *WNT5A* correlates with *IL1B* and *CCL2* (MCP-1) expression in GC tissues (Li et al., 2014). Mechanistically, Wnt5a promotes IL-1β and TNF-α expression in the GC cell line BGC-803, as well as *CCL2* expression in GC cell lines and increased macrophage migration in Transwell assays (Li et al., 2014). The link between Wnt5a and inflammation is well established (reviewed by Pashirzad et al., 2017). Therefore, a feedback mechanism might ensue in the event of inflammation, whereby early inflammatory cues induce Wnt5a expression, which in turn leads to the secretion of inflammatory chemokines, sustaining continued expression of these signals and leading to increased tumorigenicity.

**FIGURE 1:**
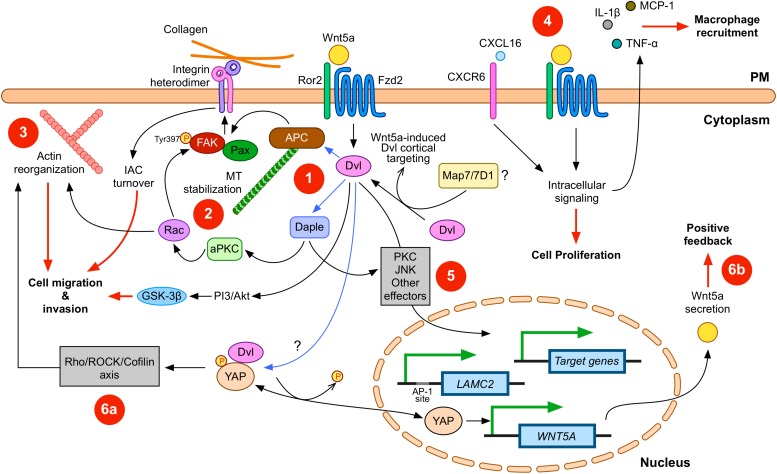
Wnt5a signaling in gastric cancer (GC). Wnt5a interacts with APC and Daple to modulate integrin adhesion complex (IAC) turnover **(1)**; this effect involves microtubule (MT) stabilization **(2)** and actin reorganization **(3)**. Disheveled (Dvl) plays a central role in this signaling axis. In addition, other signals, such as CXCL16, can cooperate with Wnt5a. Wnt5a also correlates with increased expression of pro-inflammatory cytokines **(4)**. Wnt5a might also induce changes in gene expression **(5)**. For instance, *LAMC2* is induced by Wnt5a, but other target genes might be also modulated. Finally, other signaling pathways might cooperate with Wnt5a signaling. Considering the evidence summarized here, YAP/TAZ signaling likely interacts with Wnt5a. This interaction might include direct modulation of actin remodeling **(6a)**, as well as direct induction of *WNT5A* expression after YAP dephosphorylation **(6b)**. Please note that Wnt5a might activate only some of these routes during specific stages along GC cell transformation. In addition, Dvl might be involved in all the signaling routes induced by Wnt5a. Physical interactions between Dvl and other proteins are indicated with blue arrows; biological effects are indicated by red lines. PM, plasma membrane. Frizzled-2 (Fzd2) and Ror2 are shown; however, other co-receptors and Frizzled members are likely involved. Some players and interactions are omitted for simplicity. See the main text for more details.

Collectively, these reports suggest a model for Wnt5a signaling in GC. Kikuchi and coworkers (Kikuchi et al., 2012) have proposed a model where initial epithelial cell transformation might be followed by the acquisition of mesenchymal traits due to epithelial-to-mesenchymal transition, likely due to pathways other than the non-canonical Wnt branch. In turn, transformed cells gain the ability to invade the underlying stroma. In this scenario, Wnt5a likely plays a role in promoting IAC turnover and the invasive capacity of GC cells. It is possible to expand this model to include other roles of Wnt5a, such as those related to gene expression and inflammation. An expanded overview for Wnt5a signaling, including possible roles described in this review, is depicted in Figure 1.

This model strongly supports the notion of Wnt5a expression as a negative prognosis factor in GC. However, limited evidence also suggests the opposite role for Wnt5a. Zhang et al. (2015) reported that the downregulation of Wnt5a is required for EGF-induced EMT in the GC cell line SGC-7901. In this report, the knockdown of Wnt5a increased cell migration, while the overexpression of Wnt5a impaired EGF-induced N-Cadherin and Vimentin expression. Mechanistically, Arf6 was required for EGF-induced EMT and downregulation of Wnt5a expression. In turn, the downregulation of Wnt5a expression required nuclear translocation of phosphorylated ERK and its binding to the *WNT5A* promoter (Zhang et al., 2015). In a subsequent report, it was shown that knockdown of Arf6 abrogated cell migration and invasion in the SGC-7901 cell line *in vitro*, strengthening a negative role of Wnt5a in this cell line (Qiu et al., 2018). On the contrary, Wnt5a induces EMT in the MKN-7 GC cell line (Kanzawa et al., 2013).

How can these contradictory findings be reconciled? Several explanations might be proposed. Interestingly, when Zhang et al. (2015) analyzed the effect of Wnt5a on EMT marker expression, the overexpression of Wnt5a seemed to have little or no effect. Of note, the report shows that the SGC-7901 cell line already express high levels of Wnt5a. On the other hand, Kanzawa and coworkers used MKN-7 cells, which showed the lowest expression of Wnt5a among a panel of five GC cell lines (Kanzawa et al., 2013). Therefore, the observed discrepancy might be explained by the relative expression of Wnt5a among GC cell lines. The SGC-7901 cell line also differs from other GC cell lines in terms of the expression of Wnt-related proteins, such as soluble Frizzled receptor protein 1 (SFRP1; Zhao et al., 2007) and Wls (Zhang et al., 2017). Therefore, different GC cell lines may exhibit different responses to Wnt proteins, depending on specific Wnt signaling contexts.

Finally, it remains possible that the role of Wnt5a in restricting EMT might be a specific feature of the SGC-7901 cell line. Zheng and colleagues did not corroborate their findings in other GC cell lines; meanwhile, several reports have shown the promotion of cell invasion by Wnt5a in many GC cell lines, as described in this review. Furthermore, Zheng and colleagues failed to assess the effects of EGF treatments on cell invasion in the context of Wnt5a, and most experiments were limited to morphological changes and EMT marker expression. Another interesting possibility is that Wnt5a expression must be abolished in earlier phases of EMT, but its expression might be required in later stages to promote cell migration and invasion. Notwithstanding, these findings posit a caution note when selecting cell lines for studies addressing the role of Wnt ligands in GC.

**FIGURE 2:**
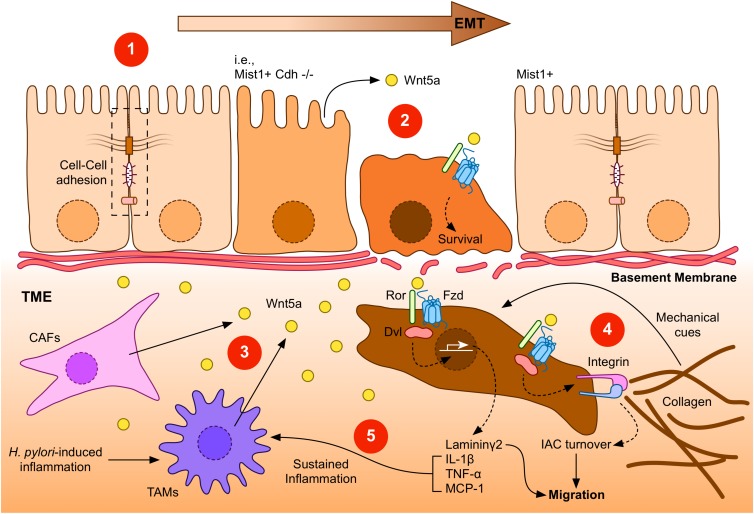
A general overview of Wnt5a signaling during GC. **(1)** Gastric epithelial cells might undergo epithelial-to-mesenchymal transition (EMT) in response to specific signals, such as loss of E-Cadherin expression (indicated as Mist1+ Cdh–/–). Transformed cells lose their epithelial characteristics, such as cell-cell interactions (dashed box). **(2)** Low levels of Wnt5a might be expressed by gastric epithelial cells. This source of Wnt5a might induce cell survival. **(3)** After the breakage of the basement membrane, transformed gastric cancer cells might find additional sources of Wnt5a, particularly from cancer-associated fibroblasts (CAFs) or tumor-associated macrophages (TAMs). In turn, increased levels of Wnt5a might signal through Frizzled (Fzd) receptors and co-receptors, such as Ror1/2 (Ror), to induce gene expression, integrin adhesion turnover, and other biological effects. **(4)** Integrins bind collagen in the tumor microenvironment (TME), forming integrin adhesion complexes (IACs). Increased turnover of IACs induced by Dvl-dependent Wnt5a signaling might promote migration and invasion of GC cells. In addition, the tumor microenvironment might provide mechanical cues, such as extracellular matrix stiffening. **(5)** Laminin γ2, which is expressed after Wnt5a stimulation, might also cooperate to promote enhanced invasiveness. Wnt5a can also induce the expression of pro-inflammatory molecules, leading to a sustained inflammatory environment, further stimulating Wnt5a secretion. *Helicobacter pylori* infection can also contribute to this environment, by promoting Wnt5a secretion.

In summary, it is possible to distinguish between Dvl-mediated mechanisms, leading to IAC turnover, and other mechanisms that might be less dependent on Dvl, which might lead to modulation of the GC environment. The former likely requires the known receptor machinery for Wnt5a, including Frizzled-2 and Ror2; however, the latter might require alternative or additional receptors and intracellular effectors. Wnt5a signaling mechanisms might be also classified as those modulating the cytoskeleton (Figure 1, left half) or those modulating other cellular processes (Figure 1, right half). In addition, these roles for Wnt5a might either overlap or represent a multi-step process, with Wnt5a playing different roles during EMT (Figure 2).

## Future Directions and Conclusion

Although the evidence discussed above strongly supports a role for Wnt5a in gastric cancer development, further research is still needed to fully understand the proper function of this Wnt ligand in the context of GC *in vivo*. Current evidence suggests that the mechanical properties of the extracellular matrix can play a significant role in cancer establishment and progression (reviewed by Pickup et al., 2014). Increased stiffness of the extracellular matrix (ECM) surrounding the tumor (usually referred to as the tumor microenvironment, or TME) can influence critical aspects of cancer cell biology, such as gene expression, invasion, and chemoresistance (Northey et al., 2017). Therefore, it would be interesting to study the mechanical changes in gastric tissues in GC and to elucidate the precise role of Wnt5a in conditions mimicking the elasticity of gastric tissue *in vivo*.

In this regard, gastric adenocarcinomas are usually preceded by mucosal atrophy and dysplasia (Lauwers and Srivastava, 2007). In addition, peritoneal dissemination (PD) is a common manifestation of GC and is associated with poor prognosis (Kanda and Kodera, 2016). PD is usually described as a multi-step process, where GC cells must invade the gastric wall and reach the peritoneal cavity (Kanda and Kodera, 2016). Carneiro and Lauwers have noted that stiffening of the gastric wall is observed during invasive adenocarcinoma, due to the desmoplasia following tumor cell invasion (Carneiro and Lauwers, 2012).

Stiffening of the ECM is partly explained by collagen cross-linking due to the action of lysyl oxidase (LOX) enzymes (Barker et al., 2012), and existing evidence suggests that LOX proteins might also play a role in GC progression (reviewed by Añazco et al., 2016). In line with these observations, Yashiro and coworkers reported that conditioned medium from the GC cell line OCUM-2MD3 induced peritoneal fibrosis in mice (Yashiro et al., 1996). Moreover, OCUM-2MD3 cells showed enhanced tumorigenicity in mice with peritoneal fibrosis induced by conditioned medium, illustrating the relevance of fibrosis for GC dissemination.

Therefore, mechanical cues from a more rigid TME might influence early steps of GC initiation, as well as later stages during PD and metastasis. The response of cancer cell lines from several tissues to mechanical cues has been characterized. However, GC cell lines have been somewhat less studied in this context, and most reports addressing the role of Wnt5a at the cellular level have been performed using GC cell lines cultured in a rigid context. Nevertheless, some limited evidence is available for GC cells. For instance, Jabbari et al. (2015) studied the behavior of AGS cells encapsulated in PEG diacrylate (PEGDA) gels, reporting that these cells are indeed sensitive to the stiffness of the PEGDA. Moreover, Branco da Cunha et al. (2016) reported a sensitivity of GC cell lines to RGD-coupled alginate hydrogels with varying stiffness. Consequently, GC cells might be responsive to mechanical cues from the TME, thus suggesting the need to evaluate such responses.

On the other hand, proteomic studies have addressed the composition of IACs in response to mechanical tension, highlighting changes in the variety and abundance of adhesome components (Humphries et al., 2009; Kuo et al., 2011; Schiller et al., 2011; Horton et al., 2015). In this regard, and given the role of Wnt5a in the turnover of focal adhesions in GC cells, it will be of interest to analyze the composition of IACs in GC cells cultured on substrates with elasticities like those found *in vivo* in gastric cancer, and to confirm if Wnt5a does modulate IAC turnover in these mechanical contexts. Tunable three-dimensional cultures of stomach organoids (McCracken et al., 2014) might be greatly useful to confirm this point.

In addition, recent evidence highlights the role of specific signaling pathways and proteins, such as YAP/TAZ (Dupont et al., 2011), in mediating cellular responses to mechanical cues. YAP and TAZ are especially interesting in this context since both proteins have been related to Wnt signaling. Initial studies focused on the role of YAP/TAZ in the context of β-catenin and Disheveled (Varelas et al., 2010; Azzolin et al., 2012, 2014; Imajo et al., 2012; Park et al., 2015). Studies have also related YAP and TAZ to cancer (reviewed by Moroishi et al., 2015; Zanconato et al., 2016), including in the gastrointestinal tract (for an updated review, see Li et al., 2019), although most articles focus on intestinal cancer (for instance, see Llado et al., 2015; Oudhoff et al., 2016; Diamantopoulou et al., 2017). However, a growing body of evidence indicates that YAP and TAZ also play a role in gastric cancer. YAP expression is commonly observed in GC samples and GC cell lines, and its expression and nuclear localization correlate with poor prognosis (Kang et al., 2011; Song et al., 2012; Zhang et al., 2012). Relevantly, YAP expression in the GC cell line MKN-28 modulates actin remodeling and promotes cell migration, while the loss of YAP induces cell stiffening and impaired migration (Qiao et al., 2017). Mechanistically, YAP directly modulates the expression of ARHGAP29, a Rho-GAP that suppresses the RhoA/LIMK/Cofilin axis. Therefore, YAP signaling via this axis results in the destabilization of actin, leading to increased cell migration (Figure 1). On the other hand, high expression of TAZ has also been detected in GC (Yue et al., 2014; Melucci et al., 2018; Wei et al., 2019). Interestingly, Melucci and coworkers evaluated the association between YAP/TAZ expression and localization, and the presence of mutations in three Wnt pathway genes (*CTNNB1*, *APC*, and *FBXW7*) in 86 patients with advanced GC. The authors reported a significant association between nuclear TAZ and mutations in the studied Wnt genes, a signature that the authors linked to increased risk of progression and reduced overall survival (Melucci et al., 2018).

Considering the role of Dvl in the context of adhesion dynamics in GC, it is of great interest to study a possible link between Wnt5a, Dvl, and YAP/TAZ in GC. Some evidence suggests potential cooperativity between these proteins in the context of tumorigenesis. Dvl physically interacts with phosphorylated YAP, promoting YAP nuclear export, and suppressing its transcriptional activity, while treatment with Wnt1 or Wnt3a induces YAP dephosphorylation, reducing its binding to Dvl and thus promoting its nuclear translocation (Lee et al., 2018). Given that Wnt5a also induces YAP dephosphorylation (Park et al., 2015), Wnt5a might also activate YAP by abrogating its binding to Dvl, thus promoting YAP nuclear translocation. In addition, TAZ also interacts with Dvl (Varelas et al., 2010). Since *WNT5A* is also a YAP/TAZ-TEAD target gene (Park et al., 2015), YAP/TAZ activation might induce Wnt5a expression, leading to positive feedback (Figure 1, bottom right). Therefore, YAP and Wnt5a might promote cell migration by two parallel mechanisms: by activating the RhoA/ROCK/LIMK/Cofilin axis to promote stress fiber dynamics, and by promoting IAC turnover, respectively. Collectively, these data suggest that YAP/TAZ and Wnt signaling components can cooperate in the establishment of GC, thus highlighting the need for further studies focused on YAP/TAZ/Wnt5a signaling in GC.

Finally, the findings reviewed in this article raise interest in possible pharmacological approaches. Several drugs have been tested in clinical trials to modulate the Wnt pathway, but most abrogate either general Wnt ligand secretion or β-catenin activity (Harb et al., 2019). Therefore, molecules specifically targeting Wnt5a signaling are needed. However, and given the multiple roles of Wnt5a, clinical approaches must be carefully designed. In this regard, a better understanding of the mechanisms of Wnt5a signaling in GC might allow the development of drugs targeting specific biological interactions triggered by this ligand, and which might also be employed in other cancers where the non-canonical pathway plays a relevant role in tumor progression and metastasis (Kikuchi et al., 2012; Endo et al., 2015; Asem et al., 2016).

In summary, the evidence analyzed in this review suggests that Wnt5a plays a significant role in gastric cancer. However, whether this role is centered on transformed cells or in cells from the TME, or whether this role is predominantly focused on IAC turnover, gene expression or inflammation, are outstanding questions. Therefore, future studies should be aimed to understand these processes in the context of mechanically pertinent environments.

## Author Contributions

PA performed the literature analysis, prepared the figures, and drafted the manuscript.

## Conflict of Interest

The authors declare that the research was conducted in the absence of any commercial or financial relationships that could be construed as a potential conflict of interest.
